# The role of bacteria and mycorrhiza in plant sulfur supply

**DOI:** 10.3389/fpls.2014.00723

**Published:** 2014-12-16

**Authors:** Jacinta Gahan, Achim Schmalenberger

**Affiliations:** Department of Life Sciences, University of LimerickLimerick, Ireland

**Keywords:** sulfonate desulfurization, sulfate esters, mycorrhizal fungi, plant–microbe interactions, asf gene cluster, sulfatases, mycorrhizosphere

## Abstract

Plant growth is highly dependent on bacteria, saprophytic, and mycorrhizal fungi which facilitate the cycling and mobilization of nutrients. Over 95% of the sulfur (S) in soil is present in an organic form. Sulfate-esters and sulfonates, the major forms of organo-S in soils, arise through deposition of biological material and are transformed through subsequent humification. Fungi and bacteria release S from sulfate-esters using sulfatases, however, release of S from sulfonates is catalyzed by a bacterial multi-component mono-oxygenase system. The *asfA* gene is used as a key marker in this desulfonation process to study sulfonatase activity in soil bacteria identified as *Variovorax*, *Polaromonas*, *Acidovorax,* and *Rhodococcus*. The rhizosphere is regarded as a hot spot for microbial activity and recent studies indicate that this is also the case for the mycorrhizosphere where bacteria may attach to the fungal hyphae capable of mobilizing organo-S. While current evidence is not showing sulfatase and sulfonatase activity in arbuscular mycorrhiza, their effect on the expression of plant host sulfate transporters is documented. A revision of the role of bacteria, fungi and the interactions between soil bacteria and mycorrhiza in plant S supply was conducted.

## INTRODUCTION

**FIGURE 1:**
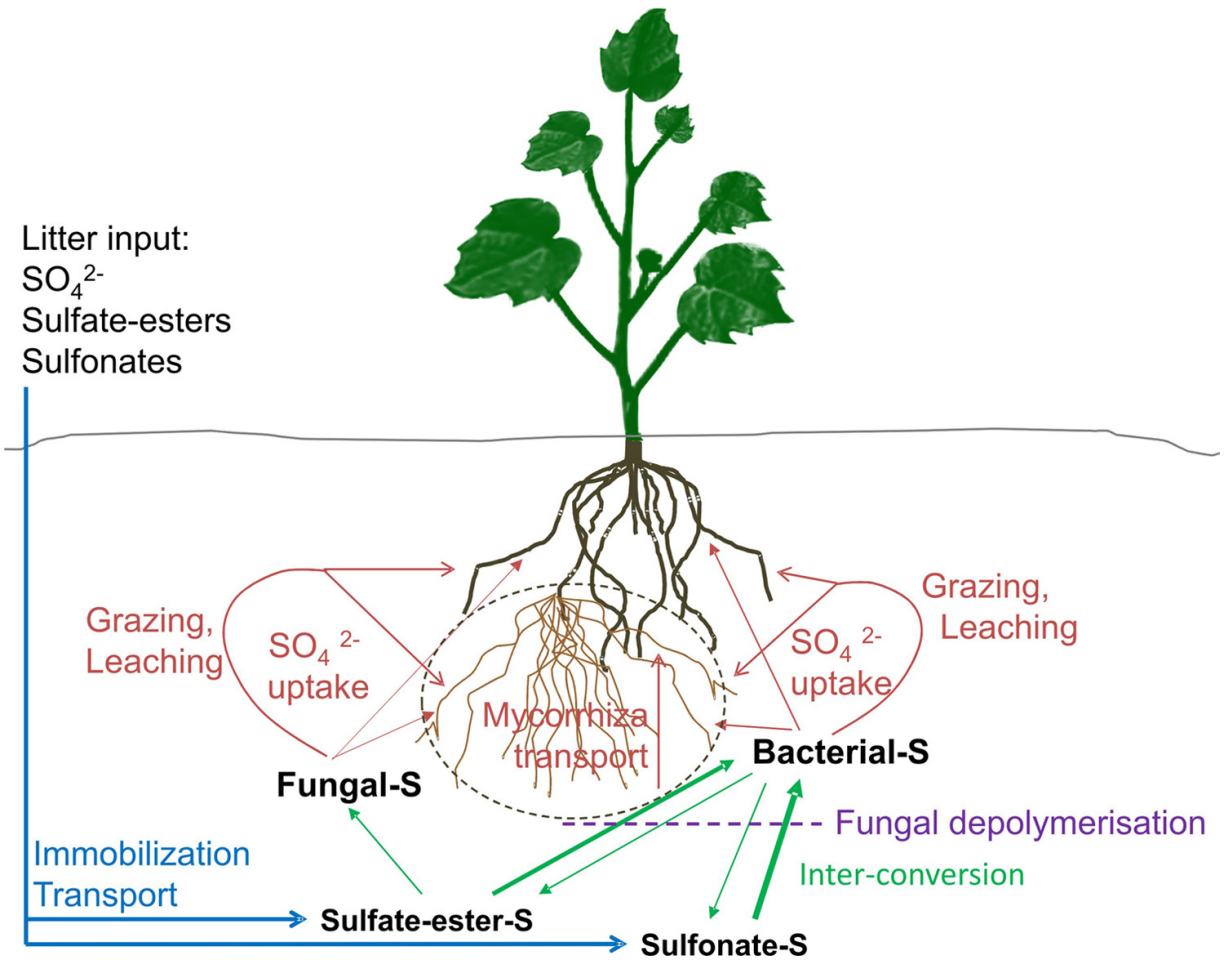
**Illustration of the sulfur cycle in soil with plant cover.** Major sulfur (S) inputs to soils originate from organic litter deposition and animal droppings (blue lines). Most of this deposited S is organically bound (organo-S). Atmospheric deposition of inorganic S has greatly declined in Europe, America and elsewhere, thus is often only a minor source for plants. Organo-S (sulfate-esters and sulfonates) can be transformed by soil microbes between the two major organo-S pools or mineralized to inorganic S (green lines, thickness suggests main direction of pathway). At the same time, inorganic S can be immobilized into organo-S (green lines). While the sulfate-ester pool is largely available to both fungi and bacteria, sulfonates are primarily accessible to bacteria only and aromatic sulfonates are only available to a particular functional clade of bacteria. Bacterial sulfonate desulfurization via the mono-oxygenase multi-enzyme pathway may occur intracellular, thus polymeric sulfonate may need depolymerisation, e.g., by saprophytic fungi prior to uptake (dotted purple line). Organo-S mineralised by fungi and bacteria need to be made available for plant uptake in the form of sulfate. This may happen via sulfate uptake by mycorrhizal fungal hyphae as an intermediate step (dashed gray line). In the absence of any direct evidence of a sulfate transport system from fungus or bacterium to the plant root or symbiotic mycorrhizal hyphae, release of mineralised S through autolysis and grazing by protists and microscopic nematodes may play an import role in inorganic sulfate release and plant sulfate uptake (red lines).

Sulfur (S), an essential macro-element required for growth, is increasingly becoming limiting to crop yield and quality as a result of a reduction in atmospheric S levels and crop varieties removing S from soil more rapidly (Fowler et al., 2005). S present in soil is approximately 95% organically bound largely in one of two major forms; sulfate-esters and sulfonates (**Figure 1**; Autry and Fitzgerald, 1990; Kertesz and Mirleau, 2004). These forms of organo-S are not directly available to plants which rely upon microbes in soil and rhizosphere for organo-S mobilization (Kertesz et al., 2007). Plant root activity impacts the physico-chemical properties of the soil through the release of organic compounds (rhizodeposition) which accounts for 15–30% of photosynthetically produced carbon (C; Russell, 1977). This process provides soil organisms with an energy source that enables them to fulfill their respective functional roles (Lynch and Whipps, 1990; Farrar et al., 2003).

Many bacteria and fungi in soil are capable of mineralizing S from sulfate-esters (Klose et al., 1999). In contrast, an exclusively bacterial multicomponent mono-oxygenase enzyme complex is necessary to mobilize sulfonates, the dominant organo-S source in soil (Vermeij et al., 1999; Kertesz and Mirleau, 2004). In fact, soil S cycling may involve complex interactions between several free living and symbiotic root associated microbial populations. Arbuscular mycorrhizal (AM) fungi form symbiosis with 80% of land plant species which depend upon them for growth (Wang and Qiu, 2006). AM fungal symbiosis is characterized by fungal penetration of root cortical cells forming microscopic branched structures called arbuscules that increase efficiency of plant-fungus metabolite exchange (Smith and Read, 1997). Extra-radicular AM hyphae provide surfaces for functional bacterial populations to colonize. A number of studies have reported interactions between AM fungi and phosphorus (P) and nitrogen (N) mobilizing bacteria (Richardson et al., 2009; Hodge and Storer, 2014), and the impact of AM on bacterial community structures (Bianciotto and Bonfante, 2002; Toljander et al., 2007). Like S, both N, and P exist predominantly inaccessible to plants which rely on interactions with mycorrhizal fungi and associated microbes to facilitate their mobilization (Richardson et al., 2009).

## SULFUR FOR PLANT GROWTH

S owes its importance as a component of the (i) proteinaceous amino acids cysteine and methionine, (ii) non-protein amino acids including cystine, lanthionine, and ethionine (iii) tripeptide glutathione, and (iv) components including vitamins thiamine and biotin, phytochelatins, chlorophyll, coenzyme A, S-adenosyl-methionin and sulfolipids (Scherer, 2001). S plays critical structural roles in cells as disulphide bonds in proteins, is involved in enzyme regulation (redox control), provides protection from oxidative stress via glutathione, and its derivatives are involved in heavy metal stress mediation (Leustek and Saito, 1999). Plant S also plays an important role in disease protection and defense response as a component of glucosinolates and allin compounds (Jones et al., 2004; Brader et al., 2006). Various plant species prevent fungal infection via deposition of elemental S in the xylem parenchyma (Cooper and Williams, 2004).

Plant S demand is dependent on species and stage of development, with increased demand observed during periods of vegetative growth and seed development (Leustek and Saito, 1999). Inorganic sulfate (${SO}_{4}^{2-}$) is the dominant plant available source of S, while to a lesser extent atmospheric reduced S may be utilized (Leustek et al., 2000). Regulation of ${SO}_{4}^{2-}$ uptake involves multiple transport steps and a large family of ${SO}_{4}^{2-}$ transporters have been characterized (Hawkesford, 2003). Assimilation of ${SO}_{4}^{2-}$ to cysteine occurs primarily in the chloroplasts of young leaves, while cysteine and methionine can also be synthesized in roots and seeds (Leustek and Saito, 1999). S starvation has been shown to negatively impact plant vitality when the P and N status is adequate (Sieh et al., 2013). During S limitation plant ${SO}_{4}^{2-}$ transporters are up-regulated for rapid ${SO}_{4}^{2-}$ up-take from the rhizosphere leading to a zone of ${SO}_{4}^{2-}$ depletion (Buchner et al., 2004). In this zone, bacterial desulfurization of organo-S is induced to mineralize organo-S, thus indirectly regulating plant S uptake (Kertesz and Mirleau, 2004). However, S-deficiency in plants can result in reduced root exudation (Alhendawi et al., 2005) or alteration of root exudates (Astolfi et al., 2010) which can influence bacterial communities seeking exudates as source of carbon.

X-ray absorption near edge structure (XANES) spectroscopy has revealed that sulfonates and sulfate-esters compose 30–70% and 20–60% of the organo-S in soil, respectively (Zhao et al., 2006). Directly plant available ${SO}_{4}^{2-}$ constitutes less than 5% of the total soil S (Autry and Fitzgerald, 1990). Organo-S compounds arise through deposition of biological material containing S, including plant and animal residues, and are subsequently incorporated into organic molecules through complex humification processes (Guggenberger, 2005). Animal residues are particularly high in organo-S with sheep dung comprising ∼80% of S as sulfonates, and while ${SO}_{4}^{2-}$ is rapidly leached from soil, organo-S can persist for longer time periods (Haynes and Williams, 1993). Additionally, soil-S pools are not static but rapidly interconverted between forms by soil microbial activity (Freney et al., 1975; Kertesz et al., 2007). Sulfonates were found to be mineralized more rapidly than other S-fractions and accounted for the majority of S released in short term incubation studies (Zhao et al., 2003, 2006). These findings indicate that C-bound S in soils may be of greatest importance (Ghani et al., 1992).

## MICROBIAL MINERALIZATION OF ORGANO-S

Microbial mineralization of organo-S is undertaken to access carbon, energy or S, with the latter also vital for plant growth (Ghani et al., 1992; Cook et al., 1998; Cook and Denger, 2002). Sulfate-ester mineralization is catalyzed by sulfatases of the esterase class (Deng and Tabatabai, 1997). Arylsulfatase enzymes act on aromatic sulfate-esters by splitting the O-S bond while alkylsulfatase enzymes act on aliphatic sulfate-esters by splitting the C-O bond (Kertesz, 1999). Both reactions release sulfate and are common in rhizospheric soil (Kertesz and Mirleau, 2004). Bacterial arylsulfatase activity is induced during S starvation and repressed in the presence of ${SO}_{4}^{2-}$ in *Pseudomonas aeruginosa,* while in a *Streptomyces* strain, a membrane bound sulfatase was also induced independently via substrate presence (Hummerjohann et al., 2000; Cregut et al., 2013). The ability to mobilize sulfate-esters has been observed in a range of bacteria including *Pseudomonas, Klebsiella, Salmonella, Enterobacter, Serratia,* and *Comamonas* (Hummerjohann et al., 2000). Additionally, arylsulfatase activity is influenced by various external factors including soil temperature, moisture content, vegetative cover, and crop rotation (Tabatabai and Bremner, 1970).

Fungi play an important role in the rhizosphere as plant symbionts or as free living saprotrophs. Soil filamentous fungi were reported to be important in mobilization of sulfate-esters (Omar and Abd-Alla, 2000; Baum and Hrynkiewicz, 2006), where enhanced arylsulfatase activity was found under S-limiting conditions (Fitzgerald, 1976; Marzluf, 1997). Likewise, wood-rotting fungi utilized sulfate-esters and thiols from wood (Schmalenberger et al., 2011).

The most abundant organo-S source in soil is present as aliphatic or aromatic sulfonates (Autry and Fitzgerald, 1990; Zhao et al., 2006). The ability to mobilize S from aliphatic sulfonates is widespread among soil bacteria with over 90% of morphologically distinct isolates capable of C2-sulfonate utilization (King and Quinn, 1997). However, aromatic sulfonates have been shown to be of greater importance for S nutrition and the ability to mobilize these sulfonates has been associated with plant growth promotion (PGP) of tomato (Kertesz and Mirleau, 2004) and *Arabidopsis* (Kertesz et al., 2007).

**FIGURE 2:**
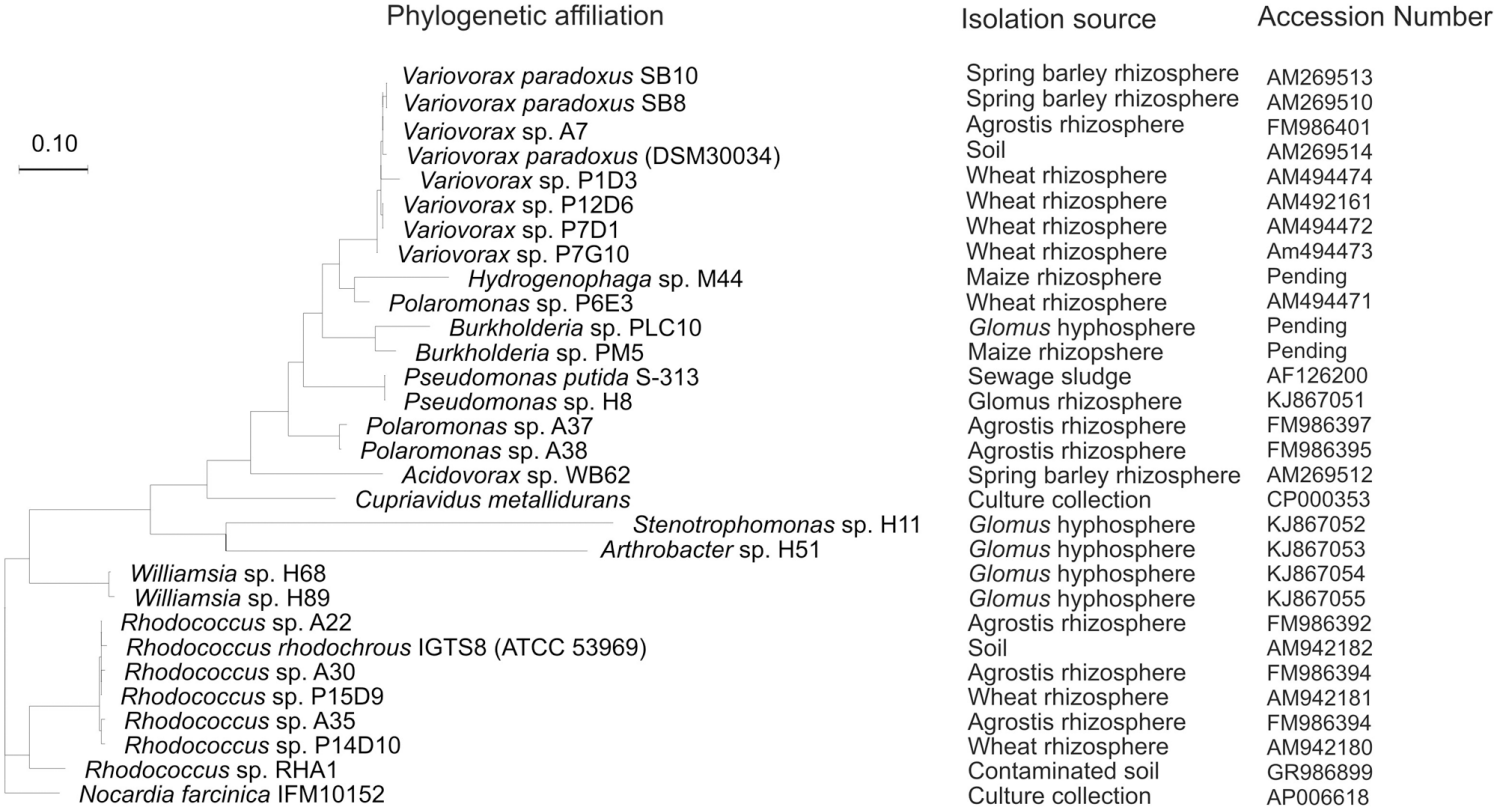
**Randomized axelerated maximum likelihood tree from truncated AsfA sequences obtained from aromatic sulfonate desulfurizing bacteria isolated from soil, rhizosphere, or hyphosphere alongside strains from culture collections**.

The desulfonating ability of the sewage sludge bacterial isolate *Pseudomonas putida* S-313 has been widely studied across a broad substrate range (Kertesz et al., 1994; Cook et al., 1998; Vermeij et al., 1999; Kahnert et al., 2000). Mobilization of ${SO}_{4}^{2-}$ from aromatic and aliphatic sulfonates is catalyzed by a FMNH_2_-dependent monooxygenase enzyme complex encoded in the *ssu* gene cluster (Eichhorn et al., 1999). The monooxygenase SsuD cleaves sulfonates to their corresponding aldehydes and the reduced flavin for this process is provided by the FMN-NADPH reductase SsuE. Although its function is unknown, *ssuF* from the *ssu* gene cluster was found to be essential for sulfonate desulfurization as well. For aromatic desulfonation the *asfRABC* gene cluster is required as an additional ‘tool-kit’ to complement *ssu*. The *asf* gene cluster includes a substrate binding protein, an ABC type transporter, a reductase/ferredoxin electron transport system involved in electron transfer and energy provision during oxygenation of the C-S bond, and a LysR-type regulatory protein, which activates the system during ${SO}_{4}^{2-}$ limitation (Vermeij et al., 1999). Transposon mutagenesis in the *asfA* gene of sewage isolate *P. putida* S-313 resulted in mutants without the capability to utilize aromatic sulfonates, while the utilization of aliphatic sulfonates was unchanged (Vermeij et al., 1999). This mutant was used in a plant growth experiment alongside its wild type, where the PGP effect was directly attributed to an functioning *asfA* gene (Kertesz and Mirleau, 2004). This particular type of bacterium has recently been isolated from the hyphae of symbiotic mycorrhizal fungi (Gahan and Schmalenberger, 2014). Various recent studies on the bacterial phylogeny of aromatic sulfonate mobilizing bacteria have expanded the diversity to the Beta-Proteobacteria; *Variovorax, Polaromonas, Hydrogenophaga, Cupriavidus, Burkholderia,* and *Acidovorax,* the Actinobacteria; *Rhodococcus* and the Gamma-Proteobacteria; *Pseudomonas* (**Figure 2**; Schmalenberger and Kertesz, 2007; Schmalenberger et al., 2008, 2009; Fox et al., 2014). Additionally, *Stenotrophomonas* and *Williamsia* species, isolated from hand-picked AM hyphae, have recently been added to these groups (Gahan and Schmalenberger, 2014).

Until now, there has been little evidence to suggest fungal catalysis of sulfonate desulfurization (Kertesz et al., 2007; Schmalenberger et al., 2011). Indeed, while some saprotrophic fungi appear to breakdown some sulfonated molecules they do not release inorganic S in the process, for example, the white rot fungus *Phanerochaete chrysporium* transforms the aromatic alkylbenzene sulfonate but does so exclusively on its side chain without S-release (Yadav et al., 2001). Cultivation of fungi *in vitro* suggested that sulfonates could be utilized as an S source by wood degrading fungus *Geophyllum trabeum*, however, XANES spectra taken from wood accessible solely to the fungus displayed no evidence of sulfonate mobilization (Schmalenberger et al., 2011). Other cultivation experiments indicated a use of aliphatic sulfonates by various strains of yeasts via a putative 2-oxoglutarate dependent dioxygenase pathway (Uria-Nickelsen et al., 1993; Linder, 2012). However, this desulfurization capability may be limited to certain C4–C6 alkanesulfonates as this is the case for the taurine dioxygenase (Kertesz, 1999). Thus, the importance of bacteria and fungi with a dioxygenase pathway for sulfonate desulfurization is still somewhat unclear. As aforementioned, bacterial desulfonation based on the monooxygenase pathway occurs intracellularly and, as such, availability of sulfonates of different molecular size may be of importance. Therefore, saprotrophic fungi, including several genera of the Basidomycota, may play a role in sulfonate mobilization by secreting enzymes such as laccases and peroxidases in order to depolymerize large organic compounds in the soil (**Figure 1**; Muralikrishna and Renganathan, 1993; Tuor et al., 1995; Heinzkill et al., 1998). Lignolytic degradation of large organic complexes releases mono and oligomeric sulfonates which can be further mobilized by functional bacterial guilds as described above (Kertesz et al., 2007).

## THE ROLE OF ARBUSCULAR MYCORRHIZA IN SULFUR SUPPLY

Arbuscular mycorrhizal fungi are the most common form of mycorrhizal association and their evolution can be dated back 460 million years (Smith and Read, 1997). They form symbiosis with 77% of angiosperms, 45% of 84 species of gymnosperms and 52% of 400 species of fern and lycopod (Wang and Qiu, 2006). The defining characteristic structure, the arbuscule, acts as an efficient site for plant-fungus metabolite exchange (Smith and Read, 1997). AM intra-radicular hyphae (IRH) provide the means for fungal extension within the host plant’s cortical region (Morton and Benny, 1990), while extra-radicular hyphae (ERH) have three primary functions – nutrient acquisition, infection of host plants, and production of fertile spores (Nagahashi and Douds, 2000).

Available studies on the effects of AM colonization on uptake of S have presented equivocal results (Gray and Gerdemann, 1973; Cooper and Tinker, 1978; Rhodes and Gerdemann, 1978). However, studies have shown that the presence of AM fungi enhances S uptake for maize, clover (Gray and Gerdemann, 1973) and tomato (Cavagnaro et al., 2006). More recently, AM fungus *G. intraradices* on transformed carrot roots demonstrated uptake of reduced forms of S *in vitro* (Allen and Shachar-Hill, 2009). Rates of this uptake and transfer of reduced S were comparable to that of ${SO}_{4}^{2-}$ when the latter was largely absent. Soil to root ${SO}_{4}^{2-}$ translocation is demand driven, with strongly induced ${SO}_{4}^{2-}$ absorption under conditions of S limitation. This rapid uptake of ${SO}_{4}^{2-}$ in the rhizosphere leads to a zone of ${SO}_{4}^{2-}$ depletion similar to that observed with P (Buchner et al., 2004). The AM fungal ERH could extend out past this zone of ${SO}_{4}^{2-}$ depletion and may play an important role in provision of S under conditions of S limitation (Kertesz et al., 2007). Recent investigations revealed that AM fungi can influence the expression of plant sulfate transporters and as a consequence improve the S nutritional status of the host plant (Giovannetti et al., 2014). This is important for all hyphospheric and rhizospheric soil microbes as lack of readily available sulfate in soil can lead to a reduction in plant exudates (Alhendawi et al., 2005) and as a consequence can affect soil microbial activity due to reduced availability of photosynthate as a source of carbon.

Extra-radicular hyphae are surrounded by complex bacterial and fungal communities that interact with the plant-mycorrhiza partnership and sustain its metabolic functioning (Frey-Klett and Garbaye, 2005). AM formation effects microbial communities in the rhizosphere via alteration of root exudates and translocation of energy rich C compounds to the extended soil environment for instance in the form of hyphal exudates (Barea et al., 2002; Boer et al., 2005). AM hyphae have a surface area several orders of magnitude greater than the plant roots which provides a niche for functional microbial interactions essential for nutrient cycling (Gryndler et al., 2000). Diverse soil microbial communities are essential for soil fertility and plant vitality (Gianinazzi and Schüepp, 1994; Siciliano et al., 2014) and AM hyphae have been shown to host a larger community of sulfonate desulfurizing bacteria than bulk soil (Gahan and Schmalenberger, 2014). Sulfonate desulfurization has been found to be characteristically rhizo- and hyphospheric in nature (**Figure 2**) and dominant sulfonate desulfurizing hyphospheric bacteria were found to be able to putatively attach and migrate with hyphae (Gahan and Schmalenberger, 2014). Inoculation of *Lolium perenne* soil microcosms with AM fungi significantly increased percentage root colonization and the quantity of cultivable sulfonate mobilizing bacteria (Gahan and Schmalenberger, 2013). Increased abundance of desulfonating bacteria as a result of elevated AM root colonization may be beneficial for plant-S supply. Likewise, addition of 2-(N-morpholine)-ethanesulfonic acid (MES) to soil putatively stimulated sulfonate mobilizing bacteria whose metabolites may have been responsible for the enhanced ERH growth of *Glomus intraradices* (Vilarino et al., 1997). This is important for maximizing S uptake as enhanced hyphal growth stemming from sulfonate mobilizing bacterial metabolites may further stimulate the proliferation of this community in a potential positive feedback loop. AM fungi may, therefore, play an increasingly important role in plant S metabolism not only through uptake and up-regulation of plant sulfate transporters but also through interaction with organo-S mobilizing microbes.

The hyphosphere of AM fungi can be regarded as a zone of increased bacterial abundance and activity, similar to the rhizosphere (Linderman, 1988; Andrade et al., 1998). Recent studies on the hyphosphere of ectomycorrhizae found that bacteria were co-migrating with the hyphae *in vitro*, putatively using a type III secretion system (T3SS) encoded infection needle for attachment (Warmink and van Elsas, 2008). This T3SS was also recently found to be present in aromatic sulfonate desulfurizing bacteria from the AM hyphosphere (Gahan and Schmalenberger, 2014), thus co-migration with ERH of AM fungi may be established via deployment of such an infection needle. While various pathogens are known to utilize T3SS for toxin injection into the host cells, nothing is known about any potential transfer of plant nutrients via such an infection needle to the mycorrhizal hyphae.

Currently, there is a profound knowledge gap when it comes to transfer of S from associated microbes to the plant host and its fungal symbiont. Extracellular sulfatases release S into soil solution which is then available to plant roots, mycorrhizal hyphae and various microbes, the release of S from sulfonates is potentially more complicated. While the possibility exists of a targeted transfer of S to the plant host via the ERH of AM fungi, there is currently no direct evidence provided in the literature. However, indirect release of S from sulfonate desulfurizing bacteria is a possibility. These bacteria may be turned over through grazing by microscopic predators such as nematodes and protozoa in the microbial loop (Bonkowski, 2004; Irshad et al., 2011). Indeed, soil amendments with biochar resulted not only in a significant increase in aromatic sulfonate desulfurizing bacteria but also in a significant increase in bacteria feeding nematodes (Fox et al., 2014), thus nematode activity may enhance the release of sulfonate desulfurized S in the rhizosphere and mycorrhizosphere/hyphosphere (**Figure 1**).

In conclusion, as a result of the limited nature of plant available S in soil it is increasingly necessary to understand the pathways and interactions required to mobilize the sulfate-esters and sulfonates that dominate the soil S pool. Saprotrophic fungi can depolymerize large humic material releasing sulfate-esters to bacteria and fungi, and sulfonates to specialist bacteria in possession of a monooxygenase enzyme complex. Desulfurizing microbial populations have been shown to be enriched in the rhizosphere and hyphosphere, however, released ${SO}_{4}^{2-}$ is quickly assimilated leaving an S depleted zone in the rhizosphere. AM fungi can extend past this zone, and indeed, are stimulated by organo-S mobilizing bacterial metabolites to expand their hyphal networks, increasing the area of soil and volume of S available to the plant. Additionally, inoculation with AM fungi has been shown to increase both percentage root colonization and the magnitude of the sulfonate mobilizing bacterial community. Inoculation practices, therefore, have huge potential to sustainably increase crop yield in areas where S is becoming a limiting factor to growth.

## Conflict of Interest Statement

The authors declare that the research was conducted in the absence of any commercial or financial relationships that could be construed as a potential conflict of interest.

## References

[B1] Alhendawi R. A. Kirkby E. A. Pilbeam D. J. (2005). Evidence that sulfur deficiency enhances molybdenum transport in xylem sap of tomato plants. *J. Plant Nutr.* 28 1347–1353 10.1081/PLN-200067449

[B2] Allen J. W. Shachar-Hill Y. (2009). Sulfur transfer through an arbuscular mycorrhiza. *Plant Physiol.* 149 549–560 10.1104/pp.108.129866 18978070 PMC2613693

[B3] Andrade G. Mihara K. Linderman R. Bethlenfalvay G. (1998). Soil aggregation status and rhizobacteria in the mycorrhizosphere. *Plant Soil* 202 89–96 10.1023/A:1004301423150

[B4] Astolfi S. Zuchi S. Hubberten H. M. Pinton R. Hoefgen R. (2010). Supply of sulphur to S-deficient young barley seedlings restores their capability to cope with iron shortage. *J. Exp. Bot.* 61 799–806 10.1093/jxb/erp346 20018904 PMC2814111

[B5] Autry A. R. Fitzgerald J. W. (1990). Sulfonate S: a major form of forest soil organic sulfur. *Biol. Fertil. Soils* 10 50–56 10.1007/BF00336124

[B6] Barea J.-M. Azcón R. Azcón-Aguilar C. (2002). Mycorrhizosphere interactions to improve plant fitness and soil quality. *Antonie Van Leeuwenhoek* 81 343–351 10.1023/A:1020588701325 12448732

[B7] Baum C. Hrynkiewicz K. (2006). Clonal and seasonal shifts in communities of saprotrophic microfungi and soil enzyme activities in the mycorrhizosphere of *Salix* spp. *J. Plant Nutr. Soil Sci.* 169 481–487 10.1002/jpln.200521922

[B8] Bianciotto V. Bonfante P. (2002). Arbuscular mycorrhizal fungi: a specialised niche for rhizospheric and endocellular bacteria. *Antonie Van Leeuwenhoek* 81 365–371 10.1023/A:1020544919072 12448735

[B9] Boer W. D. Folman L. B. Summerbell R. C. Boddy L. (2005). Living in a fungal world: impact of fungi on soil bacterial niche development. *FEMS Microbiol. Rev.* 29 795–811 10.1016/j.femsre.2004.11.005 16102603

[B10] Bonkowski M. (2004). Protozoa and plant growth: the microbial loop in soil revisited. *New Phytol.* 162 617–631 10.1111/j.1469-8137.2004.01066.x 33873756

[B11] Brader G. Mikkelsen M. D. Halkier B. A. Tapio Palva E. (2006). Altering glucosinolate profiles modulates disease resistance in plants. *Plant J.* 46 758–767 10.1111/j.1365-313X.2006.02743.x 16709192

[B12] Buchner P. Takahashi H. Hawkesford M. J. (2004). Plant sulphate transporters: co-ordination of uptake, intracellular and long-distance transport. *J. Exp. Bot.* 55 1765–1773 10.1093/jxb/erh206 15258169

[B13] Cavagnaro T. R. Jackson L. E. Six J. Ferris H. Goyal S. Asami D. (2006). Arbuscular mycorrhizas, microbial communities, nutrient availability, and soil aggregates in organic tomato production. *Plant Soil* 282 209–225 10.1007/s11104-005-5847-7

[B14] Cook A. M. Denger K. (2002). Dissimilation of the C2 sulfonates. *Arch. Microbiol.* 179 1–6 10.1007/s00203-002-0497-0 12471498

[B15] Cook A. M. Laue H. Junker F. (1998). Microbial desulfonation. *FEMS Microbiol. Rev.* 22 399–419 10.1111/j.1574-6976.1998.tb00378.x 9990724

[B16] Cooper K. M. Tinker P. (1978). Translocation and transfer of nutrients in vesicular arbuscular mycorrhizas. *New Phytol.* 88 327–339 10.1111/j.1469-8137.1981.tb01728.x

[B17] Cooper R. M. Williams J. S. (2004). Elemental sulphur as an induced antifungal substance in plant defence. *J. Exp. Bot.* 55 1947–1953 10.1093/jxb/erh179 15181110

[B18] Cregut M. Piutti S. Slezack-Deschaumes S. Benizri E. (2013). Compartmentalization and regulation of arylsulfatase activities in *Streptomyces sp., Microbacterium sp.* and *Rhodococcus sp.* soil isolates in response to inorganic sulfate limitation. *Microbiol. Res.* 168 12–21 10.1016/j.micres.2012.08.001 22921900

[B19] Deng S. Tabatabai M. (1997). Effect of tillage and residue management on enzyme activities in soils: III. Phosphatases and arylsulfatase. *Biol. Fertil. Soils* 24 141–146 10.1007/s003740050222

[B20] Eichhorn E. Van Der Ploeg J. R. Leisinger T. (1999). Characterization of a two-component alkanesulfonate monooxygenase from *Escherichia coli*. *J. Biol. Chem.* 274 26639–26646 10.1074/jbc.274.38.26639 10480865

[B21] Farrar J. Hawes M. Jones D. Lindow S. (2003). How roots control the flux of carbon to the rhizosphere. *Ecology* 84 827–837 10.1890/0012-9658(2003)084[0827:HRCTFO]2.0.CO;2

[B22] Fitzgerald J. W. (1976). Sulfate ester formation and hydrolysis: a potentially important yet often ignored aspect of the sulfur cycle of aerobic soils. *Bacteriol. Rev.* 40:698. 10.1128/br.40.3.698-721.1976 PMC413977 791238

[B23] Fowler D. Smith R. Muller J. Hayman G. Vincent K. (2005). Changes in the atmospheric deposition of acidifying compounds in the UK between 1986 and 2001. *Environ. Pollut.* 137 15–25 10.1016/j.envpol.2004.12.028 15944037

[B24] Fox A. Kwapinski W. Griffiths B. S. Schmalenberger A. (2014). The role of sulfur and phosphorus mobilizing bacteria in biochar induced growth promotion of *Lolium perenne*. *FEMS Microbiol. Ecol.* 90 78–91 10.1111/1574-6941.12374 24965962

[B25] Freney J. Melville G. Williams C. (1975). Soil organic matter fractions as sources of plant-available sulphur. *Soil Biol. Biochem.* 7 217–221 10.1016/0038-0717(75)90041-3

[B26] Frey-Klett P. Garbaye J. (2005). Mycorrhiza helper bacteria: a promising model for the genomic analysis of fungal–bacterial interactions. *New Phytol.* 168 4–8 10.1111/j.1469-8137.2005.01553.x 16159316

[B27] Gahan J. Schmalenberger A. (2013). “Bacterial and fungal communities in the mycorrhizospheres of *Agrostis*, *Lolium* and *Plantago* respond to inoculation with arbuscular mycorrhizal fungi,” in *Agricultural Research Forum 2014*, ed. Diskin M. G. (Tullamore: Teagasc), 4.

[B28] Gahan J. Schmalenberger A. (2014). Arbuscular mycorrhizal hyphae in grassland select for a diverse and abundant hyphospheric bacterial community involved in sulfonate desulfurization. *Appl. Soil Ecol.*

[B29] Ghani A. Mclaren R. Swift R. (1992). Sulphur mineralisation and transformations in soils as influenced by additions of carbon, nitrogen and sulphur. *Soil Biol. Biochem.* 24 331–341 10.1016/0038-0717(92)90193-2

[B30] Gianinazzi S. Schüepp H. (1994). *Impact of Arbuscular Mycorrhizas on Sustainable Agriculture and Natural Ecosystems.* Berlin: Birkhäuser (Springer) 10.1007/978-3-0348-8504-1

[B31] Giovannetti M. Tolosano M. Volpe V. Kopriva S. Bonfante P. (2014). Identification and functional characterization of a sulfate transporter induced by both sulfur starvation and mycorrhiza formation in *Lotus japonicus*. *New Phytol.* 204 609–619 10.1111/nph.12949 25132489

[B32] Gray L. Gerdemann J. (1973). Uptake of sulphur-35 by vesicular-arbuscular mycorrhizae. *Plant Soil* 39 687–689 10.1007/BF00264184

[B33] Gryndler M. Hršelová H. Stříteská D. (2000). Effect of soil bacteria on hyphal growth of the arbuscular mycorrhizal fungus *Glomus claroideum*. *Folia Microbiol.* 45 545–551 10.1007/BF02818724 11501421

[B34] Guggenberger G. (2005). “Humification and mineralization in soils,” in *Microorganisms in Soils: Roles in Genesis and Functions*, eds Buscot F. Varma A. (Berlin: Springer), 85–106 10.1007/3-540-26609-7_4

[B35] Hawkesford M. J. (2003). Transporter gene families in plants: the sulphate transporter gene family—redundancy or specialization? *Physiol. Plant.* 117 155–163 10.1034/j.1399-3054.2003.00034.x

[B36] Haynes R. Williams P. (1993). Nutrient cycling and soil fertility in the grazed pasture ecosystem. *Adv. Agron.* 49 119–199 10.1016/S0065-2113(08)60794-4

[B37] Heinzkill M. Bech L. Halkier T. Schneider P. Anke T. (1998). Characterization of laccases and peroxidases from wood-rotting fungi (family Coprinaceae). *Appl. Environ. Microbiol.* 64 1601–1606. 9572923 10.1128/aem.64.5.1601-1606.1998 PMC106202

[B38] Hodge A. Storer K. (2014). Arbuscular mycorrhiza and nitrogen: implications for individual plants through to ecosystems. *Plant Soil* (in press) 10.1007/s11104-014-2162-1

[B39] Hummerjohann J. Laudenbach S. Rétey J. Leisinger T. Kertesz M. A. (2000). The sulfur-regulated arylsulfatase gene cluster of *Pseudomonas aeruginosa*, a new member of the cys regulon. *J. Bacteriol.* 182 2055–2058 10.1128/JB.182.7.2055-2058.2000 10715018 PMC101934

[B40] Irshad U. Villenave C. Brauman A. Plassard C. (2011). Grazing by nematodes on rhizosphere bacteria enhances nitrate and phosphorus availability to *Pinus pinaster* seedlings. *Soil Biol. Biochem.* 43 2121–2126 10.1016/j.soilbio.2011.06.015

[B41] Jones M. G. Hughes J. Tregova A. Milne J. Tomsett A. B. Collin H. A. (2004). Biosynthesis of the flavour precursors of onion and garlic. *J. Exp. Bot.* 55 1903–1918 10.1093/jxb/erh138 15234988

[B42] Kahnert A. Vermeij P. Wietek C. James P. Leisinger T. Kertesz M. A. (2000). The ssu locus plays a key role in organosulfur metabolism in *Pseudomonas putida* S-313. *J. Bacteriol.* 182 2869–2878 10.1128/JB.182.10.2869-2878.2000 10781557 PMC101997

[B43] Kertesz M. A. (1999). Riding the sulfur cycle – metabolism of sulfonates and sulfate esters in Gram-negative bacteria. *FEMS Microbiol. Rev.* 24 135–175. 10717312 10.1016/S0168-6445(99)00033-9

[B44] Kertesz M. A. Cook A. M. Leisinger T. (1994). Microbial metabolism of sulfur and phosphorus-containing xenobiotics. *FEMS Microbiol. Rev.* 15 195–215 10.1111/j.1574-6976.1994.tb00135.x 7946467

[B45] Kertesz M. A. Fellows E. Schmalenberger A. (2007). Rhizobacteria and plant sulfur supply. *Adv. Appl. Microbiol.* 62 235–268 10.1016/S0065-2164(07)62008-5 17869607

[B46] Kertesz M. A. Mirleau P. (2004). The role of microbes in plant sulphur supply. *J. Exp. Bot.* 55 1939–1945 10.1093/jxb/erh176 15181108

[B47] King J. Quinn J. (1997). The utilization of organosulphonates by soil and freshwater bacteria. *Lett. Appl. Microbiol.* 24 474–478 10.1046/j.1472-765X.1997.00062.x

[B48] Klose S. Moore J. M. Tabatabai M. A. (1999). Arylsulfatase activity of microbial biomass in soils as affected by cropping systems. *Biol. Fertil. Soils* 29 46–54 10.1007/s003740050523

[B49] Leustek T. Martin M. N. Bick J. A. Davies J. P. (2000). Pathways and regulation of sulfur metabolism revealed through molecular and genetic studies. *Ann. Rev. Plant Biol.* 51 141–165 10.1146/annurev.arplant.51.1.141 15012189

[B50] Leustek T. Saito K. (1999). Sulfate transport and assimilation in plants. *Plant Physiol.* 120 637–644 10.1104/pp.120.3.637 10398698 PMC1539218

[B51] Linder T. (2012). Genomics of alternative sulfur utilization in ascomycetous yeasts. *Microbiology* 158 2585–2597 10.1099/mic.0.060285-0 22790398

[B52] Linderman R. (1988). Mycorrhizal interactions with the rhizosphere microflora: the mycorrhizosphere effect. *Phytopathology* 78 366–371.

[B53] Lynch J. Whipps J. (1990). Substrate flow in the rhizosphere. *Plant Soil* 129 1–10 10.1007/BF00011685

[B54] Marzluf G. A. (1997). Molecular genetics of sulfur assimilation in filamentous fungi and yeast. *Annu. Rev. Microbiol.* 51 73–96 10.1146/annurev.micro.51.1.73 9343344

[B55] Morton J. B. Benny G. L. (1990). Revised classification of arbuscular mycorrhizal fungi (Zygomycetes): a new order, *Glomales*, two new suborders, Glomineae and Gigasporineae, and two new families, Acaulosporaceae and Gigasporaceae, with an emendation of Glomaceae. *Mycotaxon* 37 471–491.

[B56] Muralikrishna C. Renganathan V. (1993). Peroxidase-catalyzed desulfonation of 3, 5-dimethyl-4-hydroxy and 3, 5-dimethyl-4-aminobenzenesulfonic acids. *Biochem. Biophys. Res. Commun.* 197 798–804 10.1006/bbrc.1993.2549 8267618

[B57] Nagahashi G. Douds D. D. (2000). Partial separation of root exudate components and their effects upon the growth of germinated spores of AM fungi. *Mycol. Res.* 104 1453–1464 10.1017/S0953756200002860

[B58] Omar S. Abd-Alla M. (2000). Physiological aspects of fungi isolated from root nodules of faba bean (*Vicia faba* L.). *Microbiol.* *Res.* 154 339–347 10.1016/S0944-5013(00)80008-7 10772156

[B59] Rhodes L. Gerdemann J. (1978). Influence of phosphorus nutrition on sulfur uptake by vesicular-arbuscular mycorrhizae of onion. *Soil Biol. Biochem.* 10 361–364 10.1016/0038-0717(78)90058-5

[B60] Richardson A. E. Barea J.-M. Mcneill A. M. Prigent-Combaret C. (2009). Acquisition of phosphorus and nitrogen in the rhizosphere and plant growth promotion by microorganisms. *Plant Soil* 321 305–339 10.1007/s11104-009-9895-2

[B61] Russell R. S. (1977). *Plant Root Systems: Their Function and Interaction with Soil* London: McGraw-Hill Book Company.

[B62] Scherer H. (2001). Sulphur in crop production—invited paper. *Eur. J. Agron.* 14 81–111 10.1016/S1161-0301(00)00082-4

[B63] Schmalenberger A. Hodge S. Bryant A. Hawkesford M. J. Singh B. K. Kertesz M. A. (2008). The role of *Variovorax* and other Comamonadaceae in sulfur transformations by microbial wheat rhizosphere communities exposed to different sulfur fertilization regimes. *Environ. Microbiol.* 10 1486–1500 10.1111/j.1462-2920.2007.01564.x 18279342

[B64] Schmalenberger A. Hodge S. Hawkesford M. J. Kertesz M. A. (2009). Sulfonate desulfurization in *Rhodococcus* from wheat rhizosphere communities. *FEMS Microbiol. Ecol.* 67 140–150 10.1111/j.1574-6941.2008.00602.x 19120463

[B65] Schmalenberger A. Kertesz M. A. (2007). Desulfurization of aromatic sulfonates by rhizosphere bacteria: high diversity of the asfA gene. *Environ. Microbiol.* 9 535–545 10.1111/j.1462-2920.2006.01172.x 17222151

[B66] Schmalenberger A. Pritzkow W. Ojeda J. J. Noll M. (2011). Characterization of main sulfur source of wood-degrading Basidiomycetes by S K-edge X-ray absorption near edge spectroscopy (XANES). *Int. Biodeterior. Biodegradation* 65 1215–1223 10.1016/j.ibiod.2011.08.013

[B67] Siciliano S. D. Palmer A. S. Winsley T. Lamb E. Bissett A. Brown M. V. (2014). Soil fertility is associated with fungal and bacterial richness, whereas pH is associated with community composition in polar soil microbial communities. *Soil Biol. Biochem.* 78 10–20 10.1016/j.soilbio.2014.07.005

[B68] Sieh D. Watanabe M. Devers E. A. Brueckner F. Hoefgen R. Krajinski F. (2013). The arbuscular mycorrhizal symbiosis influences sulfur starvation responses of *Medicago truncatula*. *New Phytol.* 197 606–616 10.1111/nph.12034 23190168

[B69] Smith S. E. Read D. J. (1997). *Mycorrhizal Symbiosis.* Oxford: Academic Press.

[B70] Tabatabai M. Bremner J. (1970). Arylsulfatase activity of soils. *Crop Sci. Soc. Am. J.* 34 225–229 10.2136/sssaj1970.03615995003400020016x

[B71] Toljander J. F. Lindahl B. D. Paul L. R. Elfstrand M. Finlay R. D. (2007). Influence of arbuscular mycorrhizal mycelial exudates on soil bacterial growth and community structure. *FEMS Microbiol. Ecol.* 61 295–304 10.1111/j.1574-6941.2007.00337.x 17535297

[B72] Tuor U. Winterhalter K. Fiechter A. (1995). Enzymes of white-rot fungi involved in lignin degradation and ecological determinants for wood decay. *J. Biotechnol.* 41 1–17 10.1016/0168-1656(95)00042-O

[B73] Uria-Nickelsen M. R. Leadbetter E. R. Godchaux W. III. (1993). Sulfonate-sulfur assimilation by yeasts resembles that of bacteria. *FEMS Microbiol. Lett.* 114 73–77 10.1111/j.1574-6968.1993.tb06553.x 8293962

[B74] Vermeij P. Wietek C. Kahnert A. Wüest T. Kertesz M. A. (1999). Genetic organization of sulphur-controlled aryldesulphonation in *Pseudomonas putida* S-313. *Mol. Microbiol.* 32 913–926 10.1046/j.1365-2958.1999.01398.x 10361295

[B75] Vilarino A. Frey B. Shüepp H. (1997). MES [2-(N-morpholine)-ethane sulphonic acid] buffer promotes the growth of external hyphae of the arbuscular mycorrhizal fungus *Glomus intraradices* in an alkaline sand. *Biol. Fertil. Soils* 25 79–81 10.1007/s003740050284

[B76] Wang B. Qiu Y. L. (2006). Phylogenetic distribution and evolution of mycorrhizas in land plants. *Mycorrhiza* 16 299–363 10.1007/s00572-005-0033-6 16845554

[B77] Warmink J. A. van Elsas J. D. (2008). Selection of bacterial populations in the mycosphere of *Laccaria proxima*: is type III secretion involved. *ISME J.* 2 887–900 10.1038/ismej.2008.41 18421258

[B78] Yadav J. S. Lawrence D. L. Nuck B. A. Federle T. W. Reddy C. A. (2001). Biotransformation of linear alkylbenzene sulfonate (LAS) by *Phanerochaete chrysosporium*: oxidation of alkyl side-chain. *Biodegradation* 12 443–453 10.1023/A:1015056530264 12051650

[B79] Zhao F. Knights J. Hu Z. Mcgrath S. (2003). Stable sulfur isotope ratio indicates long-term changes in sulfur deposition in the Broadbalk experiment since 1845. *J. Environ. Qual.* 32 33–39 10.2134/jeq2003.3300 12549539

[B80] Zhao F. Lehmann J. Solomon D. Fox M. Mcgrath S. (2006). Sulphur speciation and turnover in soils: evidence from sulphur K-edge XANES spectroscopy and isotope dilution studies. *Soil Biol. Biochem.* 38 1000–1007 10.1016/j.soilbio.2005.08.013

